# Gold Nanoparticle Approach to the Selective Delivery of Gene Silencing in Cancer—The Case for Combined Delivery?

**DOI:** 10.3390/genes8030094

**Published:** 2017-03-02

**Authors:** Rita Mendes, Alexandra R. Fernandes, Pedro V. Baptista

**Affiliations:** UCIBIO, DCV, Faculdade de Ciências e Tecnologia, Universidade Nova de Lisboa, Campus de Caparica, 2829-516 Caparica, Portugal; ars.mendes@campus.fct.unl.pt

**Keywords:** gene silencing, interfering RNA, nanotechnology, gold nanoparticles, combinatory therapy

## Abstract

Gene therapy arises as a great promise for cancer therapeutics due to its potential to silence genes involved in tumor development. In fact, there are some pivotal gene drivers that suffer critical alterations leading to cell transformation and ultimately to tumor growth. In this vein, gene silencing has been proposed as an active tool to selectively silence these molecular triggers of cancer, thus improving treatment. However, naked nucleic acid (DNA/RNA) sequences are reported to have a short lifetime in the body, promptly degraded by circulating enzymes, which in turn speed up elimination and decrease the therapeutic potential of these drugs. The use of nanoparticles for the effective delivery of these silencers to the specific target locations has allowed researchers to overcome this issue. Particularly, gold nanoparticles (AuNPs) have been used as attractive vehicles for the target-specific delivery of gene-silencing moieties, alone or in combination with other drugs. We shall discuss current trends in AuNP-based delivery of gene-silencing tools, considering the promising road ahead without overlooking existing concerns for their translation to clinics.

## 1. Gene Therapy in Cancer Therapeutics

Cancer is a leading cause of death worldwide, whose numbers are expected to keep increasing over time [1]. In 2012 alone, there were 8.2 million deaths reported, corresponding to 22% of all noncommunicable disease (NCD) deaths [2]. The conventional treatment for cancer relies on surgery, in conjunction with chemotherapy and/or radiotherapy. Despite tremendous evolution and success, there have been a growing number of cancers that have become resistant to these therapies, mostly via molecular mechanisms of drug resistance, which makes developing new strategies to overcome these issues indispensable [3]. Gene therapy arises as a great promise due to its potential to treat a disease at the molecular level, such as gene silencing; mutation correction; and antiangiogenic and suicide gene therapies [3].

In this review, we will draw our attention to gene-silencing approaches in cancer, its drawbacks and barriers, and focusing on recent progresses involving nanomedicine-based approaches toward overcoming those questions hampering full translation of gene silencing to the clinics.

### Cancer-Related Targets for Gene Silencing

The uncontrollable cell proliferation and differentiation observed in tumor cells upsurges as a result of mutational and epigenetic changes [1]. The formation of cancer is triggered by mutations in two major gene types: proto-oncogenes and tumor suppressor genes (i.e., gene “drivers” of cancer). When proto-oncogenes are overexpressed or show increased activity, which results in a stimulus of proliferation signaling pathways, they become cancer-causing genes, now called oncogenes. Tumor suppressor genes are also involved in controlling cell growth and division. When inactivated, these proteins do not act as checkpoints of cell proliferation or death, which leads to uncontrolled cell division [4]. Oncogenic or overexpression mutations lead to aberrant activation of many genes, which configures the tumors’ oncogenic phenotype. By identifying these cancer-causing genes (i.e., genes involved in the genetic alterations of tumor cells), it should be possible to design selective silencing strategies to tackle cancer at its source. Gene silencing of cancer drivers should be enhanced via the design of new drugs to target these genes [5,6,7], such as silencing genes involved in tumor initiation, growth, and spreading [1,8]. Beyond that, some disease-related molecules are considered as “non-druggable” targets, since they do not have an enzymatic function or an accessible conformation to conventional drugs (e.g., proteins), where gene therapy arises as a good alternative. Until now, several preclinical studies have shown that gene silencing can inhibit not only tumor cell growth, angiogenesis, and metastasis, but also drug resistance to chemotherapeutics [1].

Over the past two decades, antisense oligonucleotides (ASO), ribozymes, and RNA interference (RNAi) mechanisms constitute the focal strategies to specifically inhibit gene expression. All of them are antisense-guided sequence-specific silencing molecules with different mechanisms and potency of gene silencing. ASOs act by hybridizing to target mRNA, blocking the target gene’s full expression by signaling its cleavage, splicing the heteronuclear RNA into mature mRNA, nuclear–cytoplasmic transport, or translation of mRNA by steric hindrance [5]. Ribozymes are RNA molecules that feature an intrinsic catalytic activity, which then selectively recognize the target mRNA, hybridize to the specific sequence, and cleave it by catalyzing the hydrolysis of the phosphodiester backbone. Finally, RNAi is a relatively new technology that has rapidly become one of the most powerful and widely used tools for sequence-specific gene silencing [1,5].

RNAi technology is a powerful tool for target identification and has been mainly used for the modulation of gene expression, either in vitro or in vivo [6]. It is an effective approach to silence specific genes by double-stranded RNA (dsRNA). Short hairpin RNA (shRNA), endogenous microRNA (miRNA), and small interfering RNA (siRNA) are the three existent methods of RNAi technology, however, due to its ease of synthesis and not requiring genome integration, siRNA is the most appropriate for drug use [6,9]. Basically, these dsRNAs are exposed to cells via transfection or endogenous expression, and they are all cleaved by an endogenous enzyme called Dicer, which process them into smaller fragments (usually 21–23 nucleotides in length), which in turn form a complex with RNA-induced silencing complexes (RISCs) [6,8]. Then, whereas the sense strand is cleaved, the antisense strand will bind to the complementary target mRNA, consequently degrading it, resulting in translational repression or target mRNA destabilization of key oncogenic pathways (for a detailed description of the process, please refer to Guo et al., Fujita et al., and Jonas et al., [6,8,10,11]). Table 1 indicates several preclinical examples of gene silencing using RNAi technology, highlighting the molecular target in cancer development.

## 2. Addressing Current Drawbacks in Gene Silencing Therapy—Focus on Nanomedicine 

The effectiveness of RNAi strongly relates to its administration and selective delivery to the target tissue/cell based on the molecular profile of the target, and still requires full understanding of its toxicity problems before its application in clinics may be effective [5].

Despite RNAi’s high specificity that does not affect the global gene expression patterns of tissues [5], it may induce off-target effects by hybridizing with similar/homologous sequences, or silencing the target gene but not in the tissue of interest [1,5,7]. Additionally, some studies reveal that siRNAs can activate interferon, which in turn increases systemic side effects. However, these side effects are dependent on siRNA concentration, sequence, and method of generation [5]. Some extrinsic factors might also affect the effectiveness of RNAi strategies, such as sequences within the mRNAs that cannot be targeted by RNAi, effective delivery of RNAi systems to their cell/tissue targets, and their instability and low bioavailability [1,5,7]. Due to their negative charge, cell uptake is also a major concern, since they do not readily cross the cellular membranes. Even when transported by endocytosis, if they do not escape from endosomes, they will be degraded in the late endosomes fused with lysosomes [1]. When systemically delivered, naked RNAi-based therapies are exposed to nuclease degradation and are preferentially accumulated in kidney and excreted into urine due nucleic acids’ small size [5].

Naked RNA sequences are reported to have a short effect and quick elimination from the body [14]. In order to surpass such a problem, preclinical studies suggest using multiple intravenous or intraperitoneal injections [14]. Another strategy has been RNAi delivery systems, such as viral, nonviral, and targeting (for a thorough review on these please refer to [1] and references therein). Viral vectors, considered as biological vectors, despite providing efficient delivery present several drawbacks related to immunogenicity, carcinogenicity, and inflammation. Nonviral delivery systems, especially using nanotechnology systems, hold great promise to overcome some of these obstacles and allow for retardation of excretion, protection against RNases, enhanced chemical stability, and effective vectorization to the target tissue [15].

### 2.1. Nanomedicine Applied to Gene Therapy

Nanomedicine is an emerging branch of nanotechnology, in which new technologies and devices are developed at the nanoscale to improve diagnosis and therapeutics [16,17]. The design, characterization, production, and application of new structures, devices, and systems is allowed due to the control and manipulation of matter (shape and size) at nanometer range [18]. These nanomaterials are radically different from their bulk state with lengths that range from 1 to 100 nm in two or three dimensions, a definition provided by American Society for Testing and Materials (ASTM) [18,19]. Nanomedicine has been primarily focused on improving traditional medicines via incorporation into new biocompatible therapeutic agents, such as nanoparticles (NPs), enabling new administration strategies and diagnostics platforms [20]. These new structures exhibit great advantages, starting with their range size and their enhanced surface area-to-volume ratio, which permits the vectorization of different biomolecules on a single platform (e.g., peptides, antibodies, small molecules, nucleic acids, drugs), whist confer targeting capability and therapeutic properties with long circulation times in blood and plasma [10,21]. Several types of NPs, such as liposomes, polymers, dendrimers, metal (e.g., gold, silver, iron oxide), and carbon, have being developed for application in cancer therapeutics [10]. Interestingly, a combination of different NPs, dendrimer-entrapped gold nanoparticles were recently used as a nanoplatform to induce gene silencing of cancer cells [22,23].

One of the major advantages of nanomedicine in cancer is that nanostructures make use of physiological aspects of tumor morphology and physiology and the surrounding tissues’ inflammation response. The tumor tissue is physiopathologically characterized by a leaky vasculature, with wider fenestrations, which facilitates the extravasation of NPs from the surrounding vessels into its interior. Along with that, a poor lymphatic drainage is also encountered in tumors tissue. As a result, this unique abnormal structure contributes to a great vessels’ permeability and the accumulation of NPs in the tumor by passive targeting. This is known as the enhanced permeability and retention (EPR) effect [24]. The passive targeting is the most frequently used mechanism to delivery anticancer nanocarriers to the tumor. The heterogeneity of tumor vasculature and the detection and uptake of particles by the reticuloendothelial system (RES) are limitations for using this strategy. One method to reduce RES uptake is the use of small molecules grafted to the surface that confer stealth properties, such as polyethylene glycol (PEG). PEGylation of NPs’ surface produces a hydrated barrier causing steric hindrance to the attachment of phagocytes [18]. Additionally, molecules and structures may be grafted on NPs and used for active and triggered targeting of drugs to the specific tissue/cell [24]. In active targeting, NPs are modified with various probes or targeting agents—such as antibodies, small molecules, or peptides—which maximizes their preferential retention within cancer cells [25]. In triggered targeting, NPs only release their contents after some external stimulus, which can be an electric or magnetic field, ultrasound, hyperthermia, or light. Though, these nanocarriers are not easily prepared and often release their payloads when unsolicited.

Nanotechnology systems for cancer treatment may be used to target tumor sites, thus improving RNAi delivery selectively to cancer cells [26]. Cationic lipids, such as Transfectam and Lipofectamine, and biocompatible polymers, are widely used for intracellular nucleic acid delivery due to their transfection efficacy and ease of large-scale production. However, these synthetic vectors feature low storage stability, lack of targeting efficacy, and limited in vivo tracking/monitoring, limiting their clinical application. On the other hand, gold nanoparticles’ (AuNPs) optimal delivery capacity as vehicles for antisense DNA and siRNA therapy has been proved, being easily internalized by cells and allowing the knockdown of gene expression [15]. Therefore, AuNPs have quite a few advantages for being used as synthetic vehicles, compared with the ones mentioned above (for more information about other NP types, please check Keles et al., Draz et al., Tabernero et al., Kanasty et al., Williford et al., and Wan et al., [15,27,28,29,30,31,32,33]).

### 2.2. Gold Nanoparticles Applied to Gene-Silencing Therapeutics

Among the wide range of NPs, noble metal NPs have gained significant attention in recent years, particularly AuNPs due to their biocompatibility and unique physical and chemical properties, including their surface plasmon resonance (SPR) [34]. SPR relies on the interaction between an electromagnetic wave and free conduction electrons existing on the AuNPs’ surface, causing them to oscillate coherently in resonance with the frequency of visible light, which results in strong magnetic fields. This phenomenon greatly enhances all the radiative (absorption and scattering) and nonradiative (conversion of absorbed light into heat) properties associated with AuNPs, making them suitable for different biological or medical modalities [35,36].

AuNPs can be easily synthesized in different sizes and shapes by numerous approaches. The easiest way for their fabrication was firstly described by Turkevich, and consists of the reduction of gold salts in the presence of a dual-role agent, sodium citrate. This agent reduces the gold ions to start forming NPs and stabilizes them as it is adsorbed onto their surface, avoiding AuNPs’ aggregation [37]. Gold nanospheres (AuNSs), nanorods (AuNRs), nanoshells (AuNShs), nanocages (AuNCgs), nanostars (AuNSts), nanoboxes (AuNbs), nanocubes (AuNCus), nanoclusters (AuNCls), nanocrystal (AuNCrs), and triangular bipyramids (AuBps) are the different described shapes that vary according with the synthesis procedure and experimental conditions. Between those, AuNSs, AuNRs, AuNShs, and AuNCgs are the most explored for biomedical applications [38]. The AuNPs’ size and shape must be selected according with their end, once their characteristics could be modified [36,37]. Likewise, the surface modification of AuNPs is an important aspect for their uptake by cells. AuNPs are able to bind thiol and amine groups, allowing their functionalization with different biomolecules for biomedical applications [18,34]. For instance, they can be loaded with therapeutic agents, such as chemicals, siRNA, and therapeutic oligonucleotides; polymeric stabilizers like PEG, which greatly increase solubility, bioavailability, and circulation times; and targeting-specific elements, including antibodies and peptides [10,21].

AuNPs are also recognized by their thermal ablation capacities for biomedical application. AuNPs may also become great photothermal therapy (PTT) agents that can convert electromagnetic radiation into heat energy owing to electron excitation and relaxation. The AuNPs’ photoexcitation at their SPR maximum wavelength results in an efficient light-to-heat conversion [18,26]. Depending on the amount of heat generated and transferred to the surroundings, it can compromise or destroy cells, whether the AuNPs are within cancer cells or on their surface [39]. In addition, tumor tissue is thought to be more hypoxic, acidic, and nutrient-deficient than normal tissues, traits that are believed to increase the sensitivity of cancer cells to heat, though the effects of thermal therapy are dependent on cancer type [40].

Regarding the functionalization of AuNPs with synthetic and biological compounds, covalent attachment via interaction between sulfur and gold (the S–Au binding) is a strong and effective method to anchor these structures on AuNPs’ surface. Specifically, for gene-silencing applications, oligonucleotides can be chemically modified, becoming thiolated oligonucleotides, whose modification does not inhibit their biological activity. On the other hand, unmodified nucleic acids, which have a strong negative charge, can bind cationic AuNPs through ionic interactions, for instance using quaternary ammonium groups for covering AuNPs’ surface, which is a special type of amine that is positively charged in the whole range of pH [27,41]. It is important to mention that the efficiency of the silencing in vitro from both approaches have huge differences [41]. More recently, it was demonstrated the spatially controlled functionalization of AuNPs using designed diblock oligonucleotides with adenines employed as anchoring moieties, instead of using the thiol modification. By using anchoring nucleotides (adenines), which are highly oxidation-resistant, the major advantages are: the synthesis cost is reduced; the longtime stability is improved; the surface density, hybridization thermodynamics, and kinetics are better regulated by varying the polyA length; and nonspecific interaction between other sequences and gold surface is eliminated [42].

AuNPs are increasingly being employed for gene therapy purposes in vitro and in preclinical animal model studies due to the high payload, low toxicity, efficient uptake, fast endosomal escape, increased half-life; efficient, specific, and selective gene silencing and transfection; and a widespread transcriptional activation of the innate immune response [43]. As described above, dendrimer-entrapped gold nanoparticles (Au DENPs) were successfully used as platform for the delivery of *vascular endothelial growth factor* (*VEGF*) or *B-cell lymphoma/leukemia 2 protein* (*BCL-2*) siRNA into a human glioma cell line [22] or *BCL-2* siRNA to a human cervical carcinoma cell line, for silencing of *luciferase* (*LUC*) reporter gene and *enhanced green fluorescent protein* (*EGFP*) gene [23]. Moreover, Cui et al. investigated dendrimer-coated AuNRs for brcaa1-shRNA delivery into MCF7 cancer cells, which led to a successful silencing of *breast cancer 1* (*BRCAA1*) gene [44]. As another example emerges, folic acid-conjugated gold nanoparticles to deliver functional siRNA was studied using RelA siRNA to silence *RELA* expression in prostate cancer cells, since this gene is one of the five gene products of NF-κB (nuclear factor-κB, a transcription factor found in many cancers) [45]. Conde et al. developed a complex Au-nanoplatform and demonstrated in vitro and in vivo the efficacy of RNAi AuNP-based nanocarrier against the *C-MYC* proto-oncogene [46]. As well, Shaat et al. designed a polyelectrolyte complex from AuNPs with a specific siRNA to target the *C-MYC* gene [47]. More recently, Cordeiro et al. proved both the suitability and the great efficiency of Au-nanobeacons for gene silencing in embryos of a fli-enhanced green fluorescence protein (fli-EGFP) transgenic zebrafish line [48].

## 3. Combinatory Approaches

Tumors are complex diseases that involve multiple pathways and successive mutations, which present a great challenge for avoiding treatment resistance [17]. Alterations of the drug target (e.g., mutation, amplification, or splice variants), alterations of drug intracellular concentration (via altered influx/efflux), alterations in upstream and downstream effectors resulting in pathway reactivation (for instance, receptor tyrosine kinase activation or other components’ mutations/amplifications), and bypass mechanisms by parallel pathway activation (through a second receptor tyrosine kinase or mutation of a parallel serine/threonine kinase) are the four main hypotheses for drug-resistance development [49]. Even with the development of new drugs, the possibility of acquiring resistance is still a problem, which means that, although there is a great initial treatment response, the disease returns a few months later due to further gained mutations [50]. Therefore, inhibition of a specific pathway may not be enough to stop tumor progression [17].

The better understanding of genetic and molecular mechanisms of tumorigenesis allows the emergence of combined therapeutics. Combining multiple drugs with different cellular and molecular targets allows for the elimination of cells that are resistant to one of the drugs in the cocktail. It is important to mention that the number of doubly or more drug-resistant mutants in a population tends to be low. Besides, the emergence of high levels of drug-resistant mutants is avoided. This set of factors greatly increases the success of the treatment, however, toxicity and undesirable drug interactions are potential complications [50]. Associating drugs with different mechanisms of action is the best way to combine them, including cytotoxic anticancer chemicals with cytostatic anticancer drugs, antimetastatic drugs, or biotherapies (e.g., antibodies, gene therapy, RNAi, etc.). Thus, a good balance between therapeutic effectiveness and toxicity might be achieved, increasing the success rate of the treatment [51]. Nowadays, in order to be applied, combined therapeutics requires close collaboration among the entire cancer care team [2]. One of the major problems concerned with combinatory therapy is the dissimilar pharmacokinetics and biodistribution of each administered drug [17]. However, as mentioned before, AuNPs allow the loading of different moieties for cancer imaging, diagnostic and therapeutic, being considered multifunctional systems [26]. So, to one AuNP can be attached multiple therapeutic agents, independent of their physicochemical properties and pharmacological behaviors [17].

**Figure 1:**
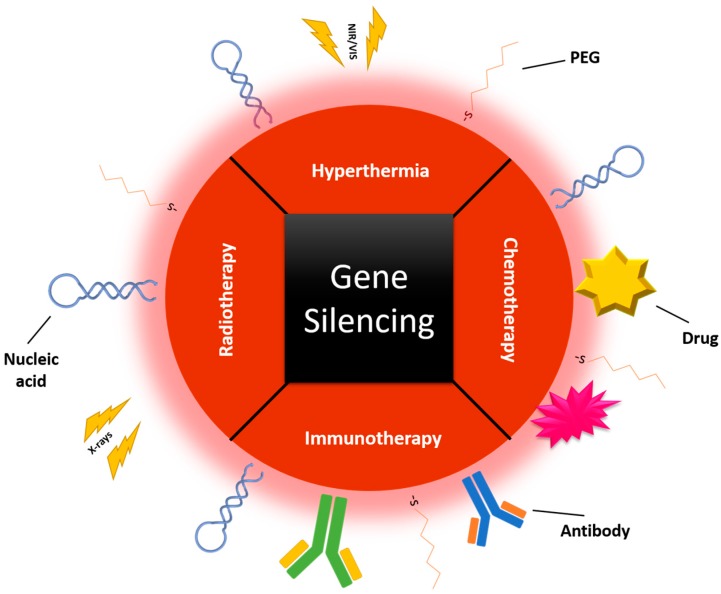
Combinatory therapy in one gold nanoparticle (AuNP), including gene silencing and other therapeutic approaches, such as chemotherapy, hyperthermia, radiotherapy, and/or immunotherapy. PEG: polyethylene glycol; NIR/VIS: Near Infra-Red/Visible.

Further therapeutic application of several combinations involving gene silencing and other treatments needs a colocalization in tumor cells of both therapies, not only for maximal synergy, but also to guarantee a minimal exposure of normal tissues to the combination. This may be achieved by using a cofunctionalization in one AuNP, or even by taking advantage of their properties, including, among others, their photothermal and radiosensitizing ability (Figure 1). Therefore, by using smart delivery systems, where combinatory products are carried together, efficacy may be enhanced without an increase to the usual deleterious side effects [52,53]. These combinatory strategies are in their infancy but their potential in cancer therapy is starting to have a real impact.

### 3.1. Gene Silencing–Gene Silencing

The combination of siRNA and miRNA, both targeting the same gene, feature potential benefits of a dual inhibition and modulation of oncogenes within the same pathway. Additionally, the combination of multiple siRNA sequences to modulate complex diseases, as cancer, has been reported and an increased therapeutic efficacy was already observed, attesting the great potential of using RNAi therapies [17,54].

The codelivery of miRNA and siRNA as cancer therapeutic agents was firstly reported by Chen et al., where an inhibition of *C-MYC*, *mouse double minute 2* (*MDM2*), and *VEGF* protein expression was performed by siRNAs in combination with a miRNA (miR-34a) to trigger cell death [55]. More recently, this strategy was tested in ovarian cancer treatment. In this case, a synergistic antitumor efficiency and greater therapeutic efficacy was achieved by a dual inhibition of *ephrin type-A receptor 2* (*EPHA2*) using a miR-520d-3p and an EphA2-siRNA. The principle presented in this approach has an extreme relevance to clinics, since Nishimura et al. believe their experimental findings can be successfully applied to other tumor-suppressive miRNAs in different human cancers [56]. On the subject of multiple-siRNA combinatory therapy, Werner et al. hypothesized a combination of a key oncogene (*K-RAS*) with an additional pathway perturbation; in this case, the apoptotic signaling pathway was chosen due to its being highly known by its equilibrium of pro- and antiapoptotic factors. With a simultaneous silencing of *B-cell lymphoma-extra-large* (*BCL-XL*), C-*FLIP*, *K-RAS*, antiapoptotic *myeloid cell leukemia-1* (*MCL1L*), *X-linked inhibitor of apoptosis protein* (*XIAP*), and *survivin* (*SUR*), they demonstrated both apoptosis induction and proliferation inhibition in pancreatic cancer cells in vitro and in vivo [57].

### 3.2. Gene Silencing–Chemotherapy

Chemotherapy is still one of the main primary treatments for cancer. Based on chemical composition and function, chemotherapy drugs can be divided into alkaline agents, which directly damage DNA, inhibiting the further division of tumor cells; antimetabolites, whose primary action is to interfere with DNA and RNA synthesis affecting DNA replication and transcription; antitumor antibiotics (anthracyclines) that also interfere with DNA replication, since they affect necessary enzymes for this process; topoisomerase inhibitors, which interfere specifically with topoisomerase, an enzyme responsible for the separation of DNA’s double stands; and mitotic inhibitors, natural products that interact and disturb the microtubule spindle machinery used in mitosis [58].

In spite of being one of the most efficient cancer treatments, mainly for more advanced stages, the development of drug resistance (primary or acquired) and the toxicity associated with these drugs are still crucial obstacles to optimal efficiency. Also, they target rapidly dividing cells, affecting not only cancerous cells but also the healthy ones, which limits the therapeutic window and the dose tolerated by patients [51,59,60,61]. Multidrug resistance (MDR) may be due to mechanisms such as reduced uptake of drugs, decreased intracellular drug concentration, modifications in cellular pathways, increased biotransformation of drugs, induced inhibition of apoptotic pathways, DNA repair mechanisms, and so forth [62]. Overexpression of drug efflux transporters is a major obstacle to chemotherapy outcome [52]. The drugs are efficiently pumped out of the tumor cells through ATP transporters, and the resistance is conferred mainly by two proteins: P-glycoprotein (P-GP, encoded by the *multidrug resistance 1* (*MDR1*) gene) and the MDR-associated proteins (MRP1) [63].

RNAi-based therapies have partial and transient antitumor effects, but when combined with chemotherapy, the result may be more potent and long-lasting [13]. Currently, there are several inhibitors against efflux activity under investigation, in order to sensitize cancer cells to chemotherapy. For instance, quinidine, cyclosporine-A, and verapamil are examples of the first-generation P-GP inhibitors; the second-generation agents include PSC833 and VX-710, and, finally, of the third-generation, tariquidar, zosuquidar, laniquidar, and ONT-093 [52]. However, one of the problems associated with these small molecules is their non-specificity, for instance, unacceptable side effects; unpredictable pharmacokinetic interactions with the anticancer drug and/or other transport proteins; and unestablished long-term safety are issues related with the first, second, and third-generation of P-GP inhibitors, respectively. For that reason, it is believed that silencing the expression of the efflux transporter rather than using an inhibitor, or even combined with it, is a better approach to overcome drug resistance [52]. Shen et al. have reported a few examples of using siRNA therapeutics targeting *MDR1* gene for sensitizing tumor cells to chemotherapeutic drugs [64]. Other possibility beyond the silencing of efflux transporter genes to enhance chemotherapy effect is silencing antiapoptotic genes, such as *BCL-2*, *heat shock proteins* (*HSPs*), *FAS*, and *P53*, since drug resistance is associated with the activation of antiapoptotic cellular defense. Most chemotherapeutic drugs induce cell death by apoptosis, which was shown to be delayed by the overexpression of *BCL-2*. Thus, by downregulation of antiapoptotic genes, cancer cells could become more sensitive to chemotherapeutic drugs [63,65].

### 3.3. Gene Silencing–Light Induced Therapeutics

Hyperthermia is a treatment that consists of the controlled increasing of body temperature or selected tissues. Nowadays, in order to localize and moderate the heat, diverse heating sources are used, for instance, radiofrequency microwaves and ultrasounds. More recently, medical procedures have been revolutionized with the discovery of lasers, which allow an even more precise thermal damage to tumor tissue [39]. Metal nanoparticles, especially AuNPs, have shown unique photophysical properties. Between these properties, the localized surface plasmon resonance (LSPR) can be enlightened. Due to this phenomenon, AuNPs, as strong visible and near infrared (NIR) light-absorption agents, have been explored as PTT agents. This has introduced a much better way to induce heating in a specific area, and also allowed the reduction of the damages in surrounding tissues, since they require lower energy doses of laser light [26,35]. AuNPs of specific sizes and shapes—including gold nanorods, nanocages, and nanoshells—have been shown as capable agents for converting NIR radiation into heat. The reason for using laser sources, whose emission is in the NIR range, is due to the negligible absorption of physiological fluids and tissues, allowing a deeper light penetration. However, using laser sources whose emission wavelength overlaps with the SPR peak of AuNPs is expected to allow a greater light-to-heat conversion. Besides, for small particles (10–40 nm in diameter), scattering is almost negligible and their extinction is almost equal to the absorption component [39].

The mechanism of cell death by photothermal effect induced by AuNPs using continuous-wave (CW) lasers in the NIR region is reported to be initiated by the disruption of the plasma membrane, followed by influx of calcium, causing membrane blebbing and damage of actin filaments, which means that laser-induced heating of AuNPs induces cell death mainly via apoptosis. Also, depending on the AuNPs’ location, the necessary laser energy will change; the energy required for AuNPs located in cytoplasm membrane is lower than for those internalized by cells [66]. In this case, as for chemotherapeutic agents, silencing antiapoptotic genes may also improve sensitivity to heat [63].

One of the major problems associated with hyperthermia is the acquisition of thermotolerance against heat stress. Thermotolerance is associated with the induction of HSPs, mainly regulated by heat shock factor 1 (HSF1). This transient resistance to additional heat stress makes it impractical to apply two different heat shocks with an interval shorter than 48–72 h, the necessary time for the resistance decays to a negligible level [67]. Because of that, one could suggests that the inhibition of the expression of *HSF1* would enhance the sensitivity to hyperthermia [68]. Recently, the key role of HSF1 was recognized to play a role in aging and oncogenesis, where its accumulation and activation is triggered by proteotoxic stress or oncogenic stress (cellular or environmental stresses associated with cancer) [69,70]. Therefore, *HSF1* arises as an attractive target for cancer therapy [70]. Studies indicate *HSF1* as a desirable target for inhibiting the stress response in cancer cells and therefore enhance the cell sensitivity to other stimuli, including hyperthermia, chemotherapy, and heavy metals. As well, correlations between high expression of HSF1 mRNA with cancer grade, metastasis, and poor prognosis suggest that it has a role in tumor progression. Several examples of the effect of silencing this gene in different cancer types have been revealing an increase of apoptosis, a reduction or inhibition of the tumor growth, a decrease in cancer cells proliferation, and an inhibition of cellular transformation and tumorigenesis, once more attesting its relevance in cancer disease [68,71,72,73].

When a strong (>50 °C) photothermal effect is used, the purpose of using hyperthermia is to increase cell death, probably by causing cell membrane disruption and protein denaturation. However, by applying a mild (43–45 °C) photothermal effect with gene delivery, a synergistic effect can be obtained. In this case, cell membrane fluidity is increased, so cellular uptake will be enhanced, facilitating endosomal escape, as well as accelerating gene release, which in turn makes gene delivery more effective.

Improved gene delivery has been also observed for AuNPs functionalized with oligonucleotides by applying a laser pulse of strong intensity, which disrupts the bonds between oligo and NP, triggering cytoplasmic release [74]. Chen et al. found that, upon irradiation, the shape and size of AuNPs changed, resulting in the release of DNA as consequence of rearrangement of the NPs’ surface at atomic level [74,75]. Another example of light-triggered gene delivery was shown by means of a bimodal treatment consisting of photothermal and photodynamic therapy combined with gene silencing [76]. In this case, ultralow doses of NIR excitation light are applied, and both effects are mediated by strong NIR light-absorbing nanomaterials (gold nanoechinus), which results in the production of reactive oxygen species (ROS) [39,76]. When this is combined with the silencing of *superoxide dismutase 1* (*SOD1*) (a very effective antiapoptotic and self-defending gene that can destroy the free radicals or reactive oxygen species) the complete destruction of deep-tissue buried tumors is promoted [76].

### 3.4. Gene Silencing–Radiotherapy

Radiotherapy is another conventional therapy, along with chemotherapy and surgery, which can be enhanced by AuNPs due to their radiosensitizing property, since gold is an excellent absorber of X-ray energy and can significantly increase the dose of absorbed irradiation and, consequently, its therapeutic effect [44,59]. The release of scattered photons and electrons following the absorption of X-rays by the tumor will induce DNA damage [44]. Combining radiotherapy with gene silencing can have a synergistic effect: on one hand, transfection/transduction efficiency, transgene integration, and the “bystander effect” of gene therapy is improved by ionizing radiation; on the other hand, the DNA susceptibility to radiation damage can be improved by gene therapy, which can also interfere with the DNA damage repair mechanisms induced by radiation. Finally, radiotherapy and gene therapy are directed at different targets in the cell cycle, thus enhancing antitumor effects [77]. The efficacy of radiotherapy is often limited by a lack susceptibility of the irradiated cells to undergo apoptosis, which is referred to as radioresistance. As mentioned before, there is an overexpression of *HSPs*, for instance *HSP70*, in several human tumors, and besides its association with a poor prognosis, it is also connected with a poor response to radiotherapy. So, transfection of cells with a siRNA against *HSP70* gene might improve the irradiation-induced apoptosis, as some in vitro studies already suggested [78].

### 3.5. Gene Silencing–Immunotherapy

The immune system is crucial to specifically obliterate cancer cells from the system, which is why immunotherapy approaches have been emerging as the next relevant tool in the clinical setting [79]. Moreover, tumor-associated immune cells are major contributors to the tumor microenvironment (TME) as well as tumor growth and development [80]. Nowadays, the focus has been mostly on tackling more advanced stages (metastasized tumors), profiting from the seek-and-destroy capability of the immune-drugs. Despite their promise, these have had limited success, thus the development of new approaches has become essential, such as enhancing the host immune system’s abilities to eliminate tumors [79,81]. Cancerous cells are recognized and their elimination is induced through immune cells, in a first line of defense via macrophages, neutrophils, and natural killer (NK) cells (innate cells), mediated through their cytotoxic mechanisms; and in a second line by macrophages and dendritic cells (DCs), which present tumor-associated antigens (TAAs) to T cells and induce a potent adaptive immune response. However, tumors develop mechanisms to evade the immune system, including changing the expression of surface molecules to become undetectable to immune cells and inducing immunotolerance by actively suppressing tumor-directed immunogenic responses (immunoediting) [80].

Gene-silencing systems allow gene-specific knockdown and also stimulate the immune system, which is independent of its primary function. This immunostimulation is related to siRNA structure and sequence, siRNA delivery system, and cell type, and it occurs when there is a link between cellular antiviral systems and the siRNA pathway [79]. In addition, intrinsic inhibitory pathways can be abolished and further immunity function can be empowered by RNAi, allowing an inhibition of immune suppression [80,81]. The combination of gene silencing with other immunotherapies can target cells from different aspects, thus possibly decreasing the tumor immune invasion. For instance, combining bortezomib, a specific inhibitor of 26S proteasome, which causes tumor cell death and also stimulates antitumor immunity, with siRNA-mediated *melanoma antigen gene member C1* (*MAGE-C1*) knockdown—MAGE-C1 is a member of cancer-testis antigens (CTAs), a group of tumor antigens—was reported to increase cell sensitivity to the first immunotherapeutic approach (bortezomib) in multiple myeloma cell lines [79]. In addition, by using gene silencing combined with short peptides to target antigens to DCs in situ have a great potential for the design of cancer vaccines, such as the combination of ipilimumab, a monoclonal antibody that blocks CTLA4, and IDO-silenced DC vaccine that was demonstrated to improve tumor immunity in a patient with melanoma [82].

## 4. Translation to the Clinics

Nanotechnology-based systems have been showing their potential as healthcare tools, especially AuNPs, one of the most studied NPs. They exhibit unique physicochemical properties and can be attached with different biomolecules, having several biomedical purposes [83]. In the last decade, the discovery of gene silencing has gained particular attention for therapeutics application. It is used to selectively turn off gene expression of abnormal overexpressed or mutated disease-associated genes, preventing their subsequent translation to proteins. The remaining challenge is the effective delivery of these gene silencing-based systems, which can be overcome using nanotechnology systems, although the success of these systems are dependent on the effective conjugation of siRNA to the nanoparticles [41,83]. Nanoparticle–siRNA conjugates are intended to enhance siRNAs circulation, promoting safe delivery to the desired location and silencing of the target mRNAs. siRNAs show tremendous sequence specificity as they can target single-nucleotide mutations, which would have an impact on selective silencing of gene isoforms and allelic specific silencing [84]. In fact, there are currently some NP-based systems for gene silencing in clinical trials, the majority of them being liposome-based (Table 2) [83,85].

Over the last 6 years, siRNA-based AuNPs have mainly been tested in cell cultures (except for one report on AuNPs for systemic administration of siRNA in humans) targeting reporter genes (e.g., luciferase or green fluorescence protein), which reveals the need for extensive testing in archetypal animal models before translation into the clinics [46,87]. Despite the great promises, the overall toxicity for both patients and environment are still concerns for AuNPs applications in the clinics [83]. These nanoconjugates have potential advantages towards delivery of therapeutics across biological barriers and compartments and in the control of the release of bioactive agents, thus enhancing therapeutic efficacy and specificity. AuNPs, due to their large surface ratio, may be multifunctional nanoplatforms, through which combinatory therapy can be accomplished [88,89] and they present unique interactions with light and radiation [37,89,90,91].

To summarize, AuNPs offer drug protection, controlled release, extended circulation, and improved targeting, featuring stimuli-responsive functions, such as photothermal and radiosensitization, whilst their impact to the environment and acute toxicity has not been critical [85,91]. The biggest challenge for the systemic delivery of Au-nanomaterials will be the demonstration of their efficient clearance/excretion, although their translation to clinics might progress quickly, compared with liposomal and monoclonal antibody technologies [92]. AuNPs are about to cross the regulatory barrier and start to be used in the clinics, focusing on gene therapy/silencing. Further developments will allow the improvement of systemic siRNA delivery combined with other approaches towards personalized medicine in cancer therapy [92].

## Figures and Tables

**Table 1:** Examples of RNA interference (RNAi)-based therapy under preclinical research.

Gene	RNAi Technology	Effect on Tumor Cells	Reference
*B-cell lymphoma/leukemia 2 protein* (*BCL-2*) (oncogene)	siRNA	Induced apoptosis in vitro	[1]
*BCL-2* (oncogene)	shRNA	Suppressed tumor growth in mice with xenograft tumor	[1]
*Clusterin* (*CLU*) (heterodimeric glycoprotein)	siRNA	Decrease in cancer cell proliferation and an increase in apoptosis rate in vitro	[1]
*CLU* (heterodimeric glycoprotein)	siRNA	*CLU* siRNA treated MDA-MB-231 cells grew significantly slower in vivo	[1]
*N-acetylglucosaminyltransferase V* (*GNT-V*) (overexpression in malignant tumors)	siRNA	Decreased proliferation of BGC823 cells	[1]
*Peptidyl-prolyl cis/trans isomerase* (*PIN1*) (overexpression in prostate and breast cancers)	shRNA	Inhibited tumor growth, metastasis and angiogenesis	[1]
*Vascular Endothelial Growth Factor* (*VEGF*)	shRNA	Inhibited cancer cell proliferation and tumor growth, and reduced tumor microvessel density (MVD) and microlymphatic vessel density (MLVD)	[1]
*Eukaryotic Initiation Factor 3 c* (*eIF3c*) (oncogene)	siRNA	The survival rate of RKO colon cancer cells drastically, the cell cycle was arrested as the number of cells entering the S phase was significantly reduced, and the induction of apoptosis was prominent	[12]
*M-BCR/ABL* fusion (constitutively activated)	siRNA	Killed leukemic cells with this arrangement	[5]
*K-RAS*^v12^ allele (oncogene)	siRNA	Specifically and stably inhibited the expression of the oncogenic *K-RAS*^v12^ allele while leaving the wild type *K-RAS* intact in human tumor cells	[5,13]

**Table 2:** Gene silencing nanoparticles (NPs) undergoing clinical trials for cancer treatment (adapted from [9,85,86]).

Name (Company)	Particle Type/Drug	Application/Indication	Clinical Trials. Gov (Phase)
TKM-080301 (Arbutus Biopharma)	Lipid particle targeting *Polo-Like Kinase 1* (*PLK1*) for delivery of siRNA	Hepatocellular carcinoma	NCT02191878 (Ph I/II)
siRNA-EphA2-DOPC (M.D. Anderson Cancer Center)	siRNA liposome for *EPHA2* knockdown	Solid tumors	NCT01591356 (Ph I)
PNT2258 (ProNAi Therapeutics)	Proprietary single-stranded DNAi (PNT100) encapsulated in lipid nanoparticles	Lymphomas	NCT02378038 (Ph II) NCT02226965 (Ph II) NCT01733238 (Ph II)
BP1001 (Bio-Path Holdings) oligonucleotide	*Growth Factor Receptor Bound Protein-2* (*GRB-2*) antisense encapsulated in neutral liposomes	Leukemia	NCT01159028 (Ph I)
DCR-MYC (Dicerna Pharmaceuticals)	DsiRNA lipid nanoparticle for *MYC* oncogene silencing	Solid tumors, multiple myeloma, lymphoma, or hepatocellular carcinoma	NCT02110563 (Ph I) NCT02314052 (Ph I/II)
Atu027 (Silence Therapeutics GmbH)	AtuRNAi^®^ liposomal formulation for *Protein Kinase N3* (*PKN3*) knockdown in vascular endothelium	Pancreatic cancer	NCT01808638 (Ph I/II)
SGT-53 (SynerGene Therapeutics)	Cationic liposome with anti-transferrin receptor antibody, encapsulating Wildtype p53 sequence	Glioblastoma, solid tumors, or pancreatic cancer	NCT02354547 (Ph I) NCT00470613 (Ph I) NCT02354547 (Ph I) NCT02340156 (Ph II)
SGT-94 (SynerGene Therapeutics)	RB94 plasmid DNA in a liposome with anti-transferrin receptor antibody	Solid tumors	NCT01517464 (Ph I)
MRX34 (Mirna Therapeutics)	Double-stranded RNA mimic of miR-34 encapsulated in liposomes	Liver cancer	NCT01829971 (Ph I)
TargomiRs (EnGeneIC)	Anti-*EGFR* bispecific antibody minicells (bacteria derived nanoparticles) with a miR-16 based microRNA payload	Mesothelioma and Non-small cell lung cancer	NCT02369198 (Ph I)
CALAA-01 (Calando Pharmaceuticals)	Polymer (targeted)/siRNA targeting *Ribonucleotide Reductase Regulatory Subunit M2* (*RRM2*)	Melanoma	NCT00689065 (Ph I)
ALN-VSP (Alnylam Pharmaceuticals)	Lipid (non-targeted)/siRNA targeting *VEGFA* and *KSP*	Solid Tumors	NCT00882180 (Ph I) NCT01158079 (Ph I)
Atu027 PKN3 (Silence Therapeutics)	Lipid (non-targeted)/siRNA targeting *PKN3*	Advanced solid tumors and metastatic pancreatic adenocarcinoma	NCT00938574 (Ph I) NCT01808638 (Ph I/II)
TKM-PLK1 (Tekmira Pharmaceuticals)	Lipid (non-targeted)/siRNA targeting *PLK1*	solid tumors, hepatocellular carcinoma, gastrointestinal neuroendocrine tumors and adrenocortical carcinoma	NCT01262235 (Ph I/II) NCT01437007 (Ph I) NCT02191878 (Ph I/II)
siG12D LODER (Silenseed)	Biodegradable polymer matrix/siRNA targeting *K-RAS*	Pancreatic Ductal Adenocarcinoma and Pancreatic cancer	NCT01188785 Ph I) NCT01676259 (Ph II)
SNS01-T (Senesco Technologies)	Polyethylenimine (non-targeted)/eIF5A^K50R^ plasmid eIF5A siRNA	NR	NCT01435720 (Ph II)

NR: Not reported.
